# A GntR Family Transcription Factor in *Streptococcus mutans* Regulates Biofilm Formation and Expression of Multiple Sugar Transporter Genes

**DOI:** 10.3389/fmicb.2018.03224

**Published:** 2019-01-14

**Authors:** Zongbo Li, Zhenting Xiang, Jumei Zeng, Yuqing Li, Jiyao Li

**Affiliations:** ^1^State Key Laboratory of Oral Diseases, National Clinical Research Center for Oral Diseases, West China Hospital of Stomatology, Sichuan University, Chengdu, China; ^2^Division of Infectious Diseases, Boston Children’s Hospital, Harvard Medical School, Boston, MA, United States

**Keywords:** GntR family transcription factor, biofilm formation, extracellular polysaccharide, *Streptococcus mutans*, global gene expression

## Abstract

GntR family transcription factors have been implicated in the regulation of carbohydrate transport and metabolism in many bacteria. However, the function of this transcription factor family is poorly studied in *Streptococcus mutans*, which is a commensal bacterium in the human oral cavity and a well-known cariogenic pathogen. One of the most important virulence traits of *S. mutans* is its ability to transport and metabolize carbohydrates. In this study, we identified a GntR transcription factor in *S. mutans* named StsR (Sugar Transporter Systems Regulator). The deletion of the *stsR* gene in *S. mutans* caused a decrease in both the formation of biofilm and the production of extracellular polysaccharides (EPS) at early stage. Global gene expression profiling revealed that the expression levels of 188 genes were changed in the *stsR* mutant, which could be clustered with the sugar PTS and ABC transporters. Furthermore, StsR protein was purified and its conserved DNA binding motif was determined using electrophoretic mobility shift assays (EMSA) and DNase I footprinting assays. Collectively, the results of this research indicate that StsR is an important transcription factor in *S. mutans* that regulates the expression of sugar transporter genes, production of EPS and formation of biofilm.

## Introduction

Dental caries is a biofilm-mediated, sugar-driven, multifactorial, dynamic disease (Pitts et al., 2017). In 2016, the top ten causes of the most prevalent diseases worldwide included caries of permanent teeth [2.44 billion, 95% UI (uncertainty interval) 2.29 billion to 2.59 billion] (GBD 2016 Disease and Injury Incidence and Prevalence Collaborators, 2017). Dental caries can occur in the presence of a cariogenic dental biofilm and frequent exposure to dietary carbohydrates, mainly free sugars (Moynihan and Kelly, 2014; Sheiham and James, 2015), which makes it a dietary-microbial disease (Zero et al., 2009). One of the crucial components of the extracellular matrix of the cariogenic oral biofilm is extracellular polysaccharides (EPS), which are mainly synthesized by *S. mutans*, a primary and well-known cariogenic pathogen(Pitts et al., 2017), through the activities of three glucosyltransferases (Gtfs) (Kawada-Matsuo et al., 2016). Therefore, the biofilm formation and EPS production abilities of *S. mutans* have been recognized as important virulence factors involved in the pathogenesis of dental caries (Xiao and Koo, 2010; Bowen and Koo, 2011).

The transport and metabolism of sugar have been shown to play an important role in the regulation of EPS synthesis, biofilm formation and virulence of *S. mutans* (Kawada-Matsuo et al., 2016). In *S. mutans*, there are two major incorporation systems involved in the transport and metabolism of sugar: the sugar phosphotransferase system (PTS) and the non-PTS system. The phosphorylated products are then catabolized to generate NADH and ATP for bacteria growth and various metabolic and acidic end products. PTS components not only participate in sugar uptake, but they also influence many other cellular processes, including biofilm formation, carbon catabolite repression (CCR), and virulence (Vadeboncoeur and Pelletier, 1997; Abranches et al., 2003; Loo et al., 2003; Abranches et al., 2006). Several genes of the PTS take part in energy and material metabolism for biofilm and the extracellular matrix, and they have been shown to significantly influence biofilm formation and EPS synthesis (Loo et al., 2003; Kawada-Matsuo et al., 2016; Zeng et al., 2017). Genes involved in the transport and metabolism of sugar in *S. mutans* are known to be regulated by transcription factors, sigma factor, two-component regulatory systems (TCS), and other proteins (Ajdic et al., 2002).

The GntR family constitutes one of the largest families of prokaryotic transcription factors (Pfam family: PF00392) (Finn et al., 2014). This transcription factor family has been studied in several bacteria, and it has been shown to regulate different genes, including various carbon metabolic genes involved in the transportation and utilization of sugar (Afzal et al., 2016; Tsypik et al., 2016, 2017). Recently, an increasing number of GntR family transcription factors have been found in various bacteria. In *Vibrio cholerae*, GntR has been reported to be involved into the Entner-Doudoroff (ED) pathway that metabolizes Gnt6P (Roy et al., 2016). In *Streptococcus pneumoniae*, the role of a GntR-family transcription factor, AgaR, has been shown to act as a transcriptional repressor of a PTS operon involved in *N*-acetylgalactosamine (NAGa) transport and utilization (Afzal et al., 2016). In *Actinobacteria*, DasR has been shown to control the transcription of genes involved in chitin and *N*-acetylglucosamine (GlcNAc) metabolism (You et al., 2018). In *Pseudomonas aeruginosa*, a GntR transcription factor has been reported to repress its own expression and the expression of the GntP gluconate permease to regulate glucose metabolism through the ED pathway (Daddaoua et al., 2017). In *S. coelicolor*, a GntR-like transcription factor of the FadR subfamily was shown to be part of a putative operon involved in gluconate metabolism (Tsypik et al., 2017).

Although the GntR family transcription factors have been studied in several bacteria, its function was poorly studied in *S. mutans*, where sugar metabolism is crucial for virulence. In *S. mutans*, seven GntR transcription factors have been reported, but their function and regulatory mechanism have not been fully elucidated yet (Ajdic et al., 2002). NagR, one of the GntR transcription factors, has been shown to be essential for the regulation of genes for both the synthesis and catabolism of glucosamine (GlcN) and *N*-acetyl-D-glucosamine (GlcNAc) in *S. mutans* (Zeng and Burne, 2015). However, the physiological function and target genes of the remaining GntR family transcription factors in *S. mutans* are still poorly understood.

In the current study, we constructed a mutant library of each GntR family transcription factor in *S. mutans*, and investigated the physiological and biochemical function of one GntR regulator, named StsR (Sugar Transporter Systems Regulator). Our results indicate that StsR influenced cell growth, biofilm formation, and EPS production in *S. mutans*. Additionally, using an in-frame mutant of *stsR*, we performed RNA-sequencing to examine possible downstream genes regulated by StsR. Using electrophoretic mobility shift assays (EMSA) and DNase I footprinting assays, the StsR DNA binding site was determined, and furthermore we found that this binding motif could be detected in the promoter regions of sugar transporters encoding operons.

## Materials and Methods

### Bacterial Strains and Growth Conditions

All bacterial strains, plasmids, and primers used in this study are listed in Supplementary Table S1. *Escherichia coli* strains were grown in Luria–Bertani medium (LB; Difco, Sparks, MD, United States). *S. mutans* UA159 and its derivatives were cultured in brain heart infusion (BHI) broth (Difco, Sparks, MD, United States) and on BHI agar or in biofilm medium (BM). For biofilm assays, BM (Solarbio, Beijing, China) was supplemented with sucrose (1%, wt/vol) as a supplementary carbohydrate source.

### Construction of In-Frame Deletion Mutants

The in-frame deletion mutant of GntR family transcription factors in *S. mutans* UA159 were constructed by a two-step transformation procedure as previously described (Xie et al., 2011). First, the *upF/upR* and *dnF/dnR* primers were used to amplify the approximately 1 kb of homologous sequence upstream and downstream of the open reading frame of the GntR family transcriptional factors, respectively. The IFDC2 cassette was amplified by PCR with the *ldhF/ermR* primers. The PCR amplicons were assembled by overlap extension PCR with the *upF/dnR* primers and then transformed into *S. mutans* UA159. To select for the transformants, 12 μg/mL erythromycin was added to the BHI plates. For the second transformation, upstream and downstream fragments of the open reading frame of the GntR family transcription factors were generated by PCR using *upF/updnR* and *dnF/dnR* primers and were overlapped to create up/down amplicon. Then, these DNA fragments were transformed into the strain obtained from the first step using BHI selection plates containing 4 mg/mL *p*-Cl-Phe (Sigma). All of the deletion mutants constructed above were further confirmed by PCR and sequencing.

### Complementation of *stsR* Mutant in Trans

The *stsR* coding sequence plus its promoter region (upstream 338 bp) was amplified with primers *SMU.1193compF* and *SMU.1193compR*, digested with *Sac*I and *Sal*I and cloned into a *Sac*I/*Sal*I digested *E. coli–Streptococcus* shuttle vector pDL278 (LeBlanc et al., 1992). The recombinant plasmid pDL278-*stsR* was then transformed into the *stsR* mutant strain. This generated the complement strain Δ*stsR*/pDL278-*stsR*, which was further confirmed by PCR and sequencing.

### Cloning, Expression, and Purification of the Recombinant StsR Protein

The *S. mutans stsR* gene coding sequence was amplified with primers *SMU.1193F* and *SMU.1193R* from *S. mutans* genomic DNA, using the high-fidelity PCR system (TakaRa). The recombinant vector pETstsR was produced by cloning the *stsR* coding sequence into the pET28a expression vector using the restriction sites listed in Supplementary Table S1. This vector was further transformed into *E. coli* BL21 (DE3) cells. After culturing the cells in 500 mL of LB medium containing 30 μg/mL kanamycin at 37°C for 5 h, isopropyl-β-D-thiogalactopyranoside (IPTG), at a final concentration of 0.3 mM, was added to the culture medium. The cells were cultured at 25°C for 5 h to induce the expression of His-StsR protein, and then StsR protein was purified as described previously (Li et al., 2010). The concentration of purified His-StsR protein was determined by measuring the spectrophotometric absorbance at 280 nm.

### Growth Curves

Overnight cultures of bacteria (1 × 10^6^ CFU/mL) grown in BHI broth were diluted 1:100 into fresh BHI broth. Bacteria were cultured in 96-well flat bottom polystyrene microtiter plates at 37°C. Cell growth was monitored with a Multiskan Spectrum (Thermo, Multiskan Go, United States), and the OD_600_ was measured in 1 h intervals. Each analysis was performed in triplicate. The representative growth curves are plotted in the figure.

For CFU counts, after diluted, the bacteria were cultured at 37°C for 1, 4, 8, and 12 h. Then bacterial suspension was serially diluted in BHI and plated on BHI agar plates. CFU values were calculated after the plates were incubated anaerobically at 37°C for 48 h. In order to see how growth looked like under different carbohydrates, the overnight bacteria (1 × 10^6^ CFU/mL) grown in BHI broth were diluted 1:100 into the tryptone-vitamin (TV) base medium (3.5% tryptone with 0.04 μg of *p*-aminobenzoic acid/ml, 0.2 μg of thiamine-HCl/ml, 1 μg of nicotinamide/ml, and 0.2 μg of riboflavin/ml) which was supplemented with either 10 mM (limiting) or 100 mM (excess) sucrose, glucose, or lactose (Moye et al., 2014c). Cell growth was monitored with a Multiskan Spectrum (Thermo, Multiskan Go, United States), and the OD_600_ was measured in 1 h intervals. Each analysis was performed in triplicate. The representative growth curves are plotted in the figure.

### Detection of Biofilm by Crystal Violet (CV) Staining

*Streptococcus mutans* UA159 and its mutants were cultured overnight and then diluted 1:100 into fresh BM supplemented with 1% (w/v) sucrose. Then, the medium was transferred to 96-well flat bottom polystyrene microtiter plates (BIOFIL, Guangzhou, China) at 100 μL per well and incubated anaerobically for 2, 4, 6, 8, 12, 24, or 48 h at 37°C under anaerobic conditions (90% N_2_, 5% CO_2_, 5% H_2_). The biofilm formation was quantified using the crystal violet (CV) staining method as previously described (Ge et al., 2008). Briefly, after biofilm formation, the medium was gently removed and the biofilm was washed with phosphate buffered saline (PBS) solution to remove planktonic bacteria. Then, the biofilms were stained with 0.4% CV and dissolved in 33% acetic acid. The absorbance at 570 nm was measured in at least triplicate for each sample. A significance of *P* < 0.05 or *P* < 0.01 was determined by Student’s *t*-test.

### Quantification of Glucans in Biofilm

*Streptococcus mutans* UA159 and mutant strains were grown in BM supplemented with 1% sucrose for 6, 12, 24, or 48 h in 24-well plates. The bacteria biofilms were collected by vortexing gently, and then they were centrifuged (10,000 *g*) for 30 min at 4°C and washed twice with ddH_2_O. To collect the water insoluble glucans (WIG), the precipitate was washed twice with 0.4 M NaOH to obtain the supernatant by centrifugation. To collect the water soluble glucans (WSG), the supernatant was mixed with 95% ethanol and maintained at 4°C for 24 h. The WIG were obtained by centrifugation. After being resuspended with 0.4 M NaOH, the supernatant was obtained by centrifugation. Every 200 μL of the WSG and WIG dissolved in the NaOH was mixed with 600 μL of the anthrone-sulfuric acid reagent. This solution mixture was maintained at 95°C for 6 min. After cooling, 200 μL of the solution was pipetted into 96-well plates, and its absorbance at 625 nm was measured. The standard curve was made according to the procedure of Laurentin and Edwards (2003). Briefly, 200 μL water (blank) and different concentrations of standard (0.05, 0.1, 0.15, 0.2, 0.25, 0.3, and 0.4 g/L glucose) were added to individual wells of a 24-well plate. The plate was vortex-mixed gently and incubated at 4°C for 15 min. Then, 600 μL of freshly prepared anthrone-sulfuric acid reagent solution was added to the plate. The solution was mixed and then maintained at 95°C for 6 min. After cooling, 200μL of this solution was pipetted into 96-well plates, and its absorbance was read at 625 nm. A linear curve was obtained within the concentration range used for the standards (0.05–0.4 g/L). The equation used was *y* = 0.3607x – 0.0793. The coefficient of determination (*R*^2^) was 0.9932, and the estimated standard deviation (s) of the regression line was 0.05. The content of WIG and WSG were determined using this equation.

### Scanning Electronic Microscopy (SEM) Analysis of Biofilm

Overnight bacterial cultures of *S. mutans* UA159 and mutant strains were adjusted to an OD_600__nm_ of 0.5, and then they were diluted 1:100 in BM supplemented with 1% sucrose. Biofilms were grown on small sterile polystyrene coverslips within 24-well flat-bottom plates at 37°C under anaerobic conditions (90% N_2_, 5% CO_2_, 5% H_2_) as mentioned above. After formation, the biofilms were washed twice using double distilled water. Then they were serially dehydrated with a graded series of ethanol, air dried, and sputter coated with gold. Samples were then observed using an SEM machine (Inspect F50; FEI, United States).

### Confocal Laser Scanning Microscopy (CLSM) Analysis of Biofilm

Biofilms were grown on coverslips, as described above. Bacterial cells and EPS from biofilms were labeled with SYTO 9 (Molecular Probes, Invitrogen, Carlsbad, CA, United States) and Alexa Fluor 647 (Molecular Probes, Invitrogen, Carlsbad, CA, United States), respectively, as previously described (Xiao et al., 2012). Biofilm images were visualized and collected by CLSM (Olympus FV1000, Japan) at a range of 495–515 nm for SYTO 9 and 655–690 nm for Alexa Fluor 647. Three-dimensional reconstruction of the biofilms with IMARIS 7.0.0 (Bitplane, Zürich, Switzerland) and the calculation of EPS/bacteria biomass were performed with Image-Pro Plus (Media Cybernetics, Silver Spring, MD, United States) and COMSTAT^1^ (Zhang et al., 2015).

### Quantitative RT-PCR

Quantitative RT-PCR was used to quantify expression of selected genes, with 16S rRNA as an internal control. *Streptococcus mutans* UA159, *S. mutans* Δ*stsR* and the complement strain Δ*stsR*/pDL278-*stsR* cells were harvested from the biofilm and snap frozen in liquid nitrogen until they were needed. Gene-specific primers were designed using the Primer3 online tools^2^ (Supplementary Table S1). Total bacterial RNA isolation, purification and reverse transcription of complementary DNA (cDNA) were performed as previously described (Li et al., 2013). Threshold cycle values (CT) were determined, and the data were analyzed by BIO-RAD CFX MANAGER software (version 2.0) using the 2^−ΔΔCT^ method.

### RNA-Seq

For transcriptome analysis, *S. mutans* UA159 and *S. mutans* Δ*stsR* cells were routinely grown at 37°C under anaerobic conditions (90% N_2_, 5% CO_2_, 5% H_2_) in BHI to an OD_600_nm of 0.5. Then they were diluted 1:100 in BM supplemented with 1% sucrose. Biofilms were grown in 6-well flat-bottom plates at 37°C under anaerobic conditions for 6 h. After formation, the biofilms were washed twice using double distilled water, and cells were collected. They were then centrifuged (4000 *g*, 4°C, 10 min) and snap frozen in liquid nitrogen until they were needed. For RNA extraction, four independent biofilm cultures of UA159 and Δ*stsR* strains were collected and treated with RNAprotect (Qiagen, Valencia, CA, United States). Total RNA was extracted and purified using RNeasy Mini kits (Qiagen) and digested with RNase-free DNase I (Qiagen). The concentration of the purified RNA samples was determined by a Nanodrop 2000 spectrophotometer (Thermo Fisher Scientific, Pittsburgh, PA, United States). cDNA libraries were constructed from enriched mRNA samples using the Truseq^TM^ RNA sample prep Kit (Illumina, San Diego, CA, United States). Isolation of rRNA from total RNA was done using Ribo-Zero Magnetic kit (Epicentre, United States) and the mRNA was chemically fragmented to short pieces using a 1× fragmentation solution (Ambion, United States) for 2.5 min at 94°C. Double stranded cDNA was produced using the SuperScript Double Stranded cDNA Synthesis Kit (Invitrogen, United States). Samples were PCR-amplified for 15 cycles with paired end primers and a randomly chosen unique barcode (Illumina, San Diego, CA, United States). RNA-seq libraries were constructed using the Illumina Paired End Sample Prep kit and sequenced using Illumina HiSeq 4000. Genes with a fold-change > 2.0 and a *P* value < 0.05 were selected for further gene expression pattern discovery. All PE reads were deposited in Sequence Read Archive database (SRA) under accession number of SRR7690952, SRR7690953, SRR7690954, SRR7690955, SRR7690956, and SRR7690957.

### Electrophoretic Mobility Shift Assays (EMSA)

DNA fragments bearing the *stsR* ORF (372 bp), *stsR* promoter (338 bp), *stsR* p1 (154 bp), *stsR* p2 (184 bp), multiple sugar-binding, ABC transporter promoter, mannitol-specific promoter, lactose-specific promoter, maltose ABC transporter promoter, and mannose-specific promoter were generated by PCR using the primers listed in Supplementary Table S1. The sugar transporter promoters contained the binding regions with StsR predicted using bioinformatical method. The 44 bp fragment *stsR* p3, containing the palindrome sequence, and the *stsR* p3mut, in which the palindrome sequence was mutated, were labeled with FITC and annealed with their reverse complement sequence. Amplified DNA fragments were extracted from agarose gels. Then, 20 pmol of DNA fragments were incubated with increasing concentrations of purified StsR in 10 μL of binding buffer (50 mM Tris/HCl, 10 mM NaCl, 0.5 M Mg/acetate, 0.1 mM EDTA, and 5% (v/v) glycerol) for 30 min. The DNA-protein complexes were resolved by electrophoresis on 4 % (w/v) non-denaturing polyacrylamide gels in 0.5× TBS buffer using the BioRad electrophoresis equipment. Gels for genes without FITC were dyed with Ethidium Bromide (EtBr) (Thermo Scientific^TM^) for 20 min. These gels were scanned by a phosphorimager and images were analyzed densitometrically using the Scion Image software (Azure Biosystems C400, United States).

### DNase I Footprinting Assays

The 184 bp fragment containing the *stsR* p2 region was amplified with specific primers labeled with FITC. The amplified products were purified with the Sigma PCR DNA purification kit (Sigma-Aldrich, St. Louis, MO, United States) and then subjected to the same binding reaction as in EMSA. DNase I footprinting was performed as previously described (Li et al., 2010). Then, reaction mixtures with 20 pmol of gene products and either 0, 20, or 40 pmol StsR protein were treated at 28°C for 3 min with DNase I (1 unit, Solarbio). The samples were phenol-extracted, ethanol precipitated, and eluted in 15 μL of distilled water. The samples were then added to the HiDi Formamide and GeneScan-500LIZ size standard. Final fragments were analyzed with an Applied Biosystems 3730XL DNA analyzer (manufactured by Tsingke Company, Chengdu). Electropherograms were analyzed and aligned using the GENEMAPPER software (Thermo Fisher Scientific, United States).

## Results

### StsR Affects the Early Stage Biofilm Formation of *S. mutans*

**FIGURE 1:**
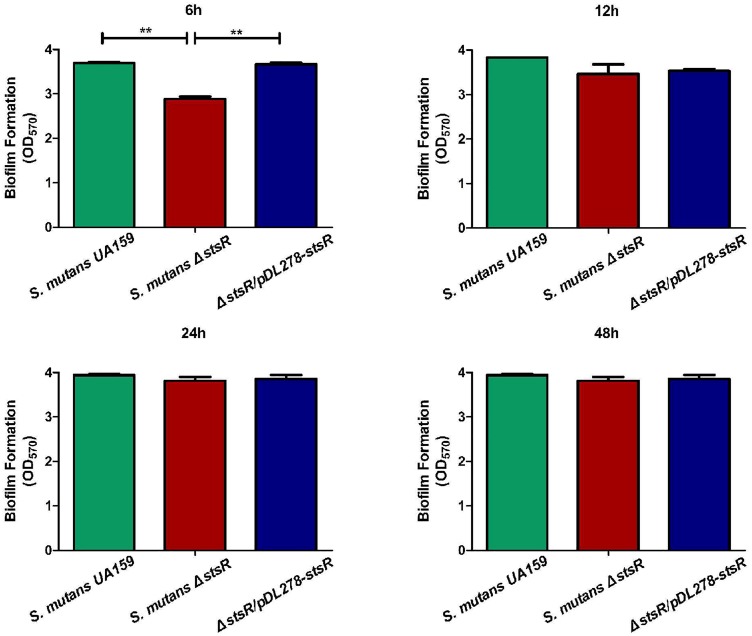
Deletion of *stsR* decreases *S. mutans* biofilm formation at early stage. *S. mutans* was cultured in BM supplemented with 1% sucrose for 6, 12, 24, and 48 h. The biofilm biomass was determined by CV staining method. Data from three biological replicates were averaged, and the statistical significance between the *stsR* mutant, wild-type, and complement strain was determined by Student’s *t*-test. Error bars represent standard deviations based on results from at least three biological replicates. ^∗∗^Indicates a significance of *P* < 0.01.

To identify the function of each GntR family transcription factor, we built a mutant library including each of the GntR family genes in *S. mutans*: *S. mutans* Δ*640c, S. mutans* Δ*953c, S. mutans* Δ*1012c, S. mutans* Δ*1064c, S. mutans* Δ*1065c, S. mutans* Δ*1193*, and *S. mutans* Δ*2040*. Biofilm formation ability of the mutants was quantified by CV staining. Compared to wild-type *S. mutans*, we found that *S. mutans* Δ*1193* had the most robust decrease in biofilm biomass (*P* < 0.01) (Supplementary Figure S1). So we choose *S. mutans* Δ*1193* for further study and named gene *S. mutans 1193* as *stsR.* To confirm this result, the *stsR*-complement strain was constructed. Biofilms of the *stsR* mutant, the wild-type, and the complement strain were further quantified by CV staining. When grown for varying times, a significant difference could be observed at 6 h, however, this difference disappeared at later stages of biofilm growth. The representative biofilm growth curves are plotted in the Supplementary Figure S2. From this figure, we found that the growth rate of the biofilm of *stsR* mutant was markedly reduced compared to the wild-type, but the final yield was almost the same. When grown for 6 h, the *stsR* mutant demonstrated a significant decrease in biofilm formation compared to the wild-type and the complement strain (*P* < 0.01). There was no observed significant difference between the biofilms of the wild-type and that of the complement strain (Figure 1). These results indicate that *stsR* could affect the formation of biofilm in *S. mutans* at early stage.

### Deletion of *stsR* Causes the Growth Delay of *S. mutans*

**FIGURE 2:**
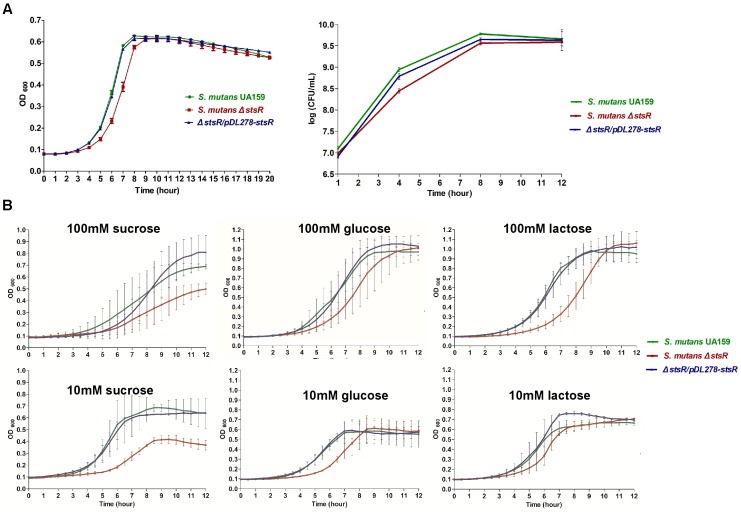
Determination of growth curves of *S. mutans* UA159, *S. mutans* Δ*stsR*, and Δ*stsR/pDL278-stsR*. *S. mutans* UA159, *S. mutans* Δ*stsR*, and Δ*stsR/pDL278-stsR* strains were cultivated in BHI to mid-exponential phase and then diluted into **(A)** fresh BHI broth or **(B)** TV base medium supplemented with either 10 mM (limiting) or 100 mM (excess) sucrose, glucose, or lactose. Growth curves were monitored with a Multiskan Spectrum (Thermo, Multiskan Go, United States), and the OD_600_ was measured in 1 h intervals. **(A)** For CFU counts, after diluted, the bacteria were cultured at 37°C for 1, 4, 8, and 12 h. Then bacterial suspension was serially diluted in BHI and plated on BHI agar plates. CFU values were calculated after the plates were incubated anaerobically at 37°C for 48 h. Error bars represent standard deviations based on results from at least three biological replicates.

The impact of *stsR* knock-out on the growth of *S. mutans* UA159 was examined by measuring the growth curves of the *stsR* mutant, wild-type, and complement strain. Significant growth inhibition was observed at about 3 h. This inhibition was observed between *stsR* mutant and both wild-type and complement strain, but there was no difference between wild-type and complement strain. Moreover, the inhibitory effect reduced with time, and at about 9 h, the difference completely disappeared (Figure 2A). As shown in Figure 2A, the growth rate of the *stsR* mutant was markedly reduced compared to the wild-type, but the final yield was almost the same. Two different methods showed the same result. These results indicate that the inactivation of *stsR* gene inhibited the growth of *S. mutants*, but it did not influence the final yield. These results were consistent with the delayed biofilm formation at early stage.

As shown in Figure 2B, when cultures were grown in TV base medium supplemented with either 10 mM (limiting) or 100 mM (excess) sucrose, glucose, or lactose, the growth curves of glucose and lactose were consistent with the results of CFU counts and growth curves when grown in BHI in Figure 2A. But when grown in sucrose supplemented medium, not only the growth rate of *S. mutans*, but also the final yield was decreased.

### Deletion of *stsR* Decreases the Quantity of Glucans in *S. mutans* Biofilm

**FIGURE 3:**
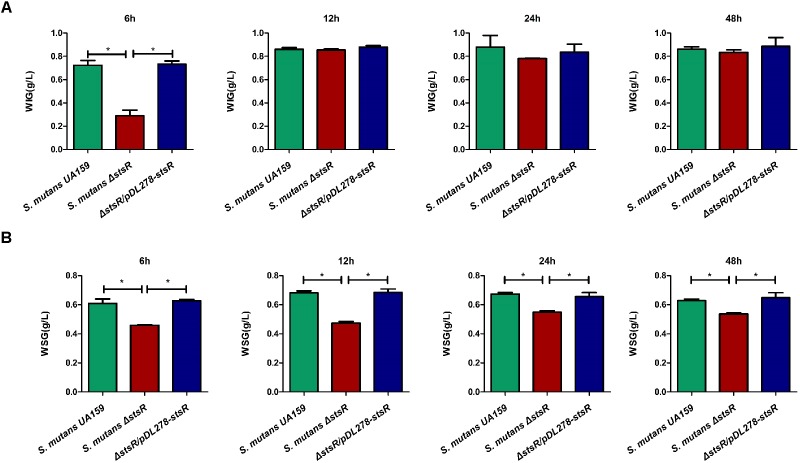
Deletion of *stsR* decreases the amount of glucans in *S. mutans* biofilm. The amounts of **(A)** water insoluble glucans and **(B)** water soluble glucans in the biofilms of *S. mutans* UA159, *S. mutans* Δ*stsR*, and complement strain were quantified using the phenol-sulfuric acid method and calculated according to the standard curve. Error bars represent standard deviations based on results from at least three biological replicates. ^∗∗^Indicates a significance of *P* < 0.01.

Glucans substantially contribute to the physical integrity and stability of oral biofilms, and they also contribute to the formation of oral cariogenic biofilm (Kawada-Matsuo et al., 2016). Previous studies have shown that the GntR family regulators regulate carbohydrate metabolism in several bacteria (Shafeeq et al., 2013; Afzal et al., 2016; Wu et al., 2016; Tsypik et al., 2017). Therefore, we measured the EPS (glucans) production by GntR mutants. To examine this, biofilms formed by wild-type and GntR mutants were scraped from polystyrene wells. The resulting solutions from the biofilms mainly contained the alkali-soluble fraction of water-insoluble glucans (WIG). As shown in Supplementary Figure S1, among the GntR family mutants, *S. mutans* Δ*1193 (stsR)*, *S. mutans* Δ*1064c*, *S. mutans* Δ*1065c*, and *S. mutans* Δ*2040* demonstrated significantly reduced abilities in synthesizing EPS. To further examine these observed differences caused by *stsR*, both water-soluble (WSG) and water-insoluble glucans (WIG) produced by wild-type, *stsR* mutant, and complement strain at 6, 12, 24, and 48 h were quantified by the phenol-sulfuric acid method. As shown in Figure 3A, the *stsR* mutant has significantly less accumulation of WIG at 6 h, as compared to both wild-type and complement strain. There was no difference observed between the wild-type and the complement strain. However, with growth time increased, the differences in WIG accumulation disappeared. For the WSG, this decreased amount was significantly lower in the *stsR* mutant until 48 h (Figure 3B).

**FIGURE 4:**
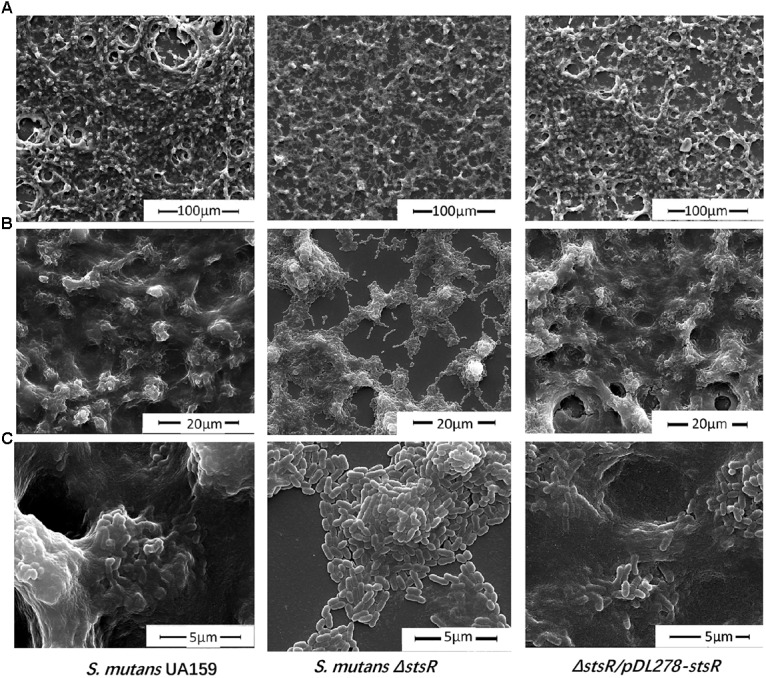
Scanning electron microscopy analysis reveals altered biofilm morphology and decreased biofilm extracellular matrix of *S. mutans* Δ*stsR*. Biofilms formed by *S. mutans* UA159, *S. mutans* Δ*stsR*, and complement strain were grown for 6 h and then scanned by scanning electron microscopy (SEM) under **(A)** 1000× magnification, **(B)** 5000× magnification, and **(C)** 20000× magnification.

**FIGURE 5:**
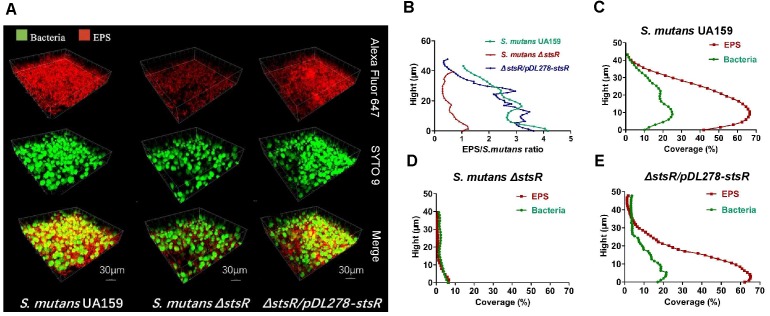
Biofilm structure and EPS distribution of *S. mutans* strains observed by confocal microscopy. **(A)** Double-labeling of 6 h *S. mutans* biofilms. Green indicates bacteria (SYTO 9), and red indicates EPS (Alexa Fluor 647). Images were taken at 60× magnification. The three-dimensional reconstruction of the biofilms and the quantification of EPS/bacteria biomass were performed with IMARIS 7.0.0. **(B)** The ratio of EPS to bacteria at different heights was quantified with COMSTAT. Results are the average of five randomly selected positions of each sample and are presented as mean ± standard deviation. **(C–E)** Quantification of *S. mutans* UA159 **(C)**, *S. mutans* Δ*stsR* **(D)**, and Δ*stsR/pDL278-stsR* **(E)** biofilms. EPS biomass was performed with COMSTAT at different heights. Results are the average of five randomly selected positions of each sample and are presented as mean ± standard deviation.

The biofilm and EPS of wild-type, *stsR* mutant, and complement strain were further examined by scanning electron microscopy (SEM) and confocal laser scanning microscopy (CLSM) to assess the changes in biofilm structure. Based the results above from the quantity of EPS, the *stsR* mutant accumulated significantly less WSG and WIG than both wild-type and complement strain at about 6 h. Thus, we chose the 6-h timepoint to observe the biofilm via microscopy. As shown in Figure 4, the loss of *stsR* resulted in decreased biofilm formation and also caused a decrease in the quantity of extracellular matrix, in which the main component was EPS. Evidently, the biofilm of the *stsR* mutant had much less EPS when scanned under a higher magnification (20,000×) (Figure 4). These results could also be obtained using CLSM. After growing for 6 h, the biofilm of the *stsR* mutant was much sparser and thinner. Furthermore, we found that *stsR* mutant cells formed biofilms with less EPS (Figure 5A). As shown in Figures 5B–E, the EPS/*S. mutans* ratio of the biofilm formed by *stsR* mutant cells was significantly lower than those of the biofilms formed by wild-type and complement strain. These results indicate that the inactivation of *stsR* inhibits the generation of glucans in *S. mutans* biofilm and results in a sparser biofilm of *stsR* mutant, when compared to wild-type and complement strain.

### Transcriptomics Analysis of *S. mutans stsR* Mutant

**FIGURE 6:**
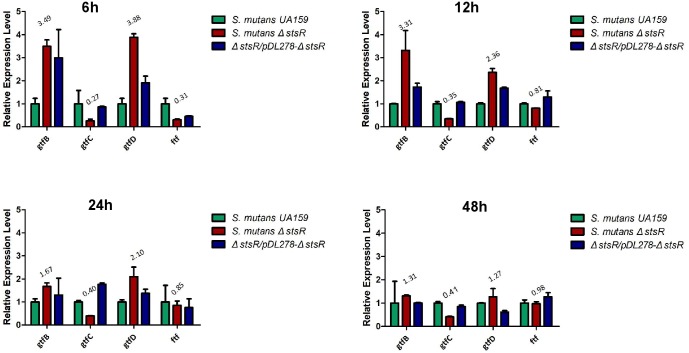
Quantitative RT-PCR assays for the relative expression levels of *gtfs* and *ftf* genes in *S. mutans* UA159, *S. mutans* Δ*stsR*, and Δ*stsR/pDL278-stsR*. The experiments were carried out as described in Experimental procedures. All target genes were amplified using specific primers. Different gene expressions were normalized to the levels of 16S rRNA gene transcripts, and the folds of expression change were calculated.

Since the deletion of *stsR* decreased the quantity of glucans in *S. mutans* biofilm, we performed the qRT-PCR to examin the expression of the *gtf* and *ftf* genes which encoded the glucosyltransferase (Gtf) and fructosyltransferase (Ftf) in the *S. mutans* biofilm at 6, 12, 24, and 48 h. As shown in the Figure 6, the expression of *gtfB* and *gtfD* were upregulated but the expression of *gtfC* and *ftf* were downregulated and all the changes of these genes were decreased at later stages of biofilm growth. Since both the WSG and WIG were significantly decreased in *S. mutans* biofilm, these results were confused. We further performed the transcriptome analysis to examine the whole gene expression in the *stsR* mutant stain.

**FIGURE 7:**
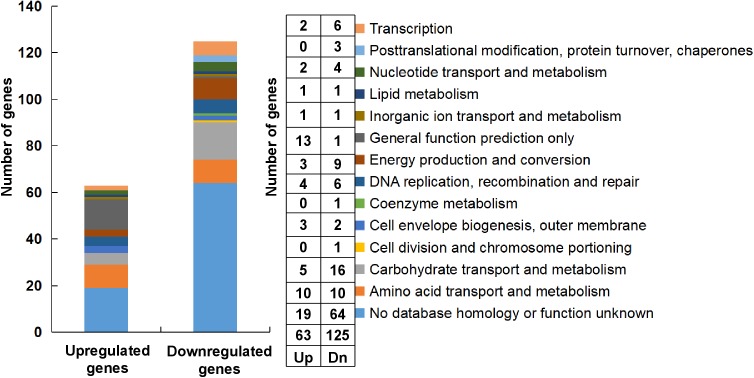
Classification and percentage of the DEGs. The classification and percentage of the DEGs were analyzed according to their functional annotations (Ajdic et al., 2002).

**FIGURE 8:**
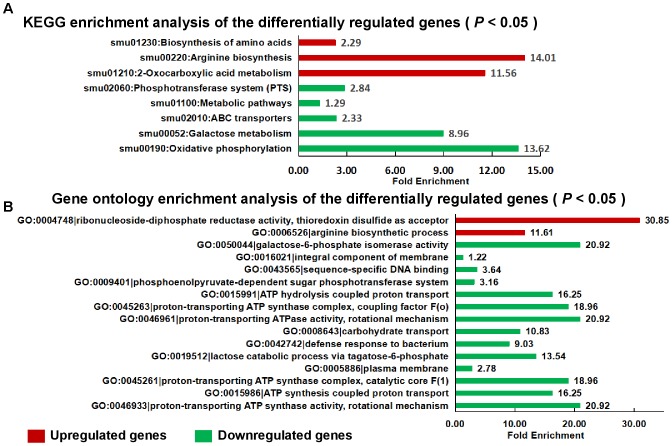
Functional categories and enrichment analysis of the DEGs. KEGG enrichment **(A)** and gene ontology **(B)** enrichment analysis of the DEGs using the DAVID tool. Upregulated genes are shown in red, and downregulated genes are shown in green. GO, gene ontology; KEGG, Kyoto Encyclopedia of Genes and Genomes.

For the transcriptome analysis, biofilm of *S. mutans* UA159 and *S. mutans* Δ*stsR* cells were grown for 6 h and collected for RNA extraction and sequencing. 188 genes were identified as differentially expressed between UA159 and the *stsR* mutant. As shown in Figure 7 and Supplementary Tables S2, S3, S6, 63 genes were significantly upregulated and 125 genes were significantly downregulated in *stsR* mutant when compared to UA159 strain. According to the National Center for Biotechnology Information (NCBI) *S. mutans* genome annotation, the function of the majority of the differentially expressed genes (DEGs) are unknown. Among the genes with changes in expression, those with known functions were mainly associated with the following processes: carbohydrate transport and metabolism; DNA replication, recombination and repair; energy production and conversion, transcription; nucleotide transport and metabolism; posttranslational modification, protein turnover, chaperones, and amino acid transport and metabolism (Figure 7). To better condense the gene lists (Supplementary Tables S2, S3) into gene functional groups, the DAVID bioinformatic tool^3^ was used to visualize many-genes-to-many-terms relationships, to cluster redundant and heterogeneous terms into groups, to search for interesting and related genes or terms, and to dynamically view genes from their lists on biopathways (Huang da et al., 2009a,b). As shown in Figure 8A and Supplementary Table S4, the DEGs were enriched in 8 Kyoto Encyclopedia of Genes and Genomes (KEGG) pathways. The downregulated genes were involved in the PTS, ABC transporters, galactose metabolism, and metabolic pathways. Similarly, in GO (Gene Ontology^4^) terms, downregulated genes primarily belong to transport systems, including the phosphoenolpyruvate-dependent sugar PTS and the carbohydrate transport system (Figure 8B and Supplementary Table S5).

**FIGURE 9:**
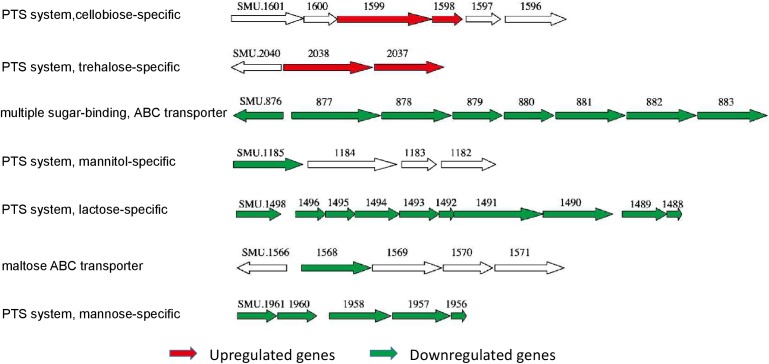
The differentially expressed sugar transport system operons in *S. mutans* Δ*stsR*. The genetic organization of differentially expressed gene clusters that were associated with sugar transport systems in *S. mutans* Δ*stsR*. Upregulated genes are colored red, and downregulated genes are colored green.

The data from the transcriptome analysis confirmed our results of the *gtfs* and *ftf* genes that the expression of *gtfB* and *gtfD* were upregulated but the expression of *gtfC* and *ftf* were downregulated but the expression levels of *gtfs* and *ftf* were not significantly changed in the *stsR* mutant strain. Instead, we found the significantly changed genes associated with sugar transporter systems. Among the gene clusters regulated by StsR, those associated with sugar metabolism could be classified into different sugar transporter systems. As shown in Figure 9, we found that StsR could regulate the expression of maltose, lactose, mannitol, mannose, cellobiose, and trehalose transporters. To validate further the reliability of our transcriptome analysis data, the expression levels of respective genes in the down regulated sugar transporters were determined by qRT-PCR. As shown in Supplementary Figure S3, the genes exhibited consistent patterns of differential expression in both the qRT-PCR and transcriptome analysis, suggesting a good concordance between both methods and conforming that genes included in the sugar transporters downregulated by StsR were significantly downregulated in the *stsR* mutant stain.

### StsR Directly Binds to Its Own Operon Promoter and the Sugar Transporter Promoters

**FIGURE 10:**
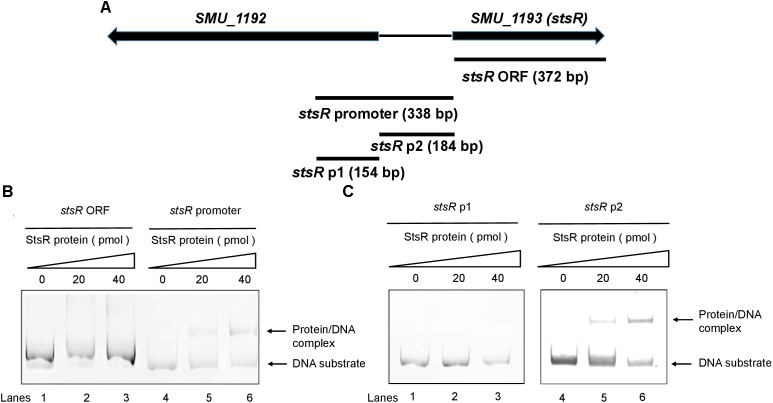
Binding of StsR to the promoter region of itself. A total of 20 pmol of DNA substrates were incubated with different amounts of StsR protein. **(A)** The location of *stsR* ORF, *stsR* promoter, *stsR* p1 and *stsR* p2 DNA substrates used in EMSA assays. **(B)** EMSA results for StsR binding to *stsR* ORF (lanes 1–3) and the *stsR* promoter (lanes 4–6). **(C)** EMSA results for StsR binding to *stsR* p1 (lanes 1–3) and the *stsR* p2 (lanes 4–6). The StsR protein level in lanes 1 and 4 was 0 pmol, lanes 2 and 5, 20 pmol, lanes 3 and 6, 40 pmol.

Transcription factors are able to regulate their own expression, which is known as feedback regulation (Mao et al., 2013; Vasanthakumar et al., 2013). Thus, we chose the promotor of *stsR* itself to study the DNA binding activity and specificity of StsR. To further investigate the interaction between StsR and the regulated promoters, a total of four gene regions were subjected to EMSA with purified His-StsR protein. Among these gene regions, the *stsR* promotor was a 319 bp gene fragment upstream the *stsR* gene ORF, and the 372 bp *stsR* gene ORF was used to exclude non-specific binding (Figure 10A). We observed shifts in mobility when StsR was incubated with the *stsR* promoter fragment, but no shift was observed when StsR was incubated with the *stsR* ORF fragment (Figure 10B). Therefore, StsR could specifically bind to the *stsR* promoter region. To more precisely identify the region that StsR interacts with, we separated the *stsR* promoter into two segments: *stsR* p2 (184 bp), contains the region between *smu.1193* and *smu.1192*, and *stsR* p1 (154 bp). As shown in Figure 10C, specific binding was observed when StsR was incubated with the *stsR* p2, but no protein/DNA complex was observed when incubated with the *stsR* p1. These results suggested that StsR could specifically bind to the *stsR* p2 region.

To further investigate the interaction between StsR and the regulated sugar transporter promoters, gene regions containing the predicted promoter sequences of sugar transporter operons determined by FIMO were subjected to EMSA with purified His-StsR protein. As shown in Supplementary Figure S4, StsR could specifically bind to the predicted promoter sequences in mannitol-specific PTS transporter, mannose-specific PTS transporter, maltose ABC transporter and multiple sugar-binding ABC transporter. But only one of the predicted promoter sequences in lactose-specific transporter PTS transporter could bind with StsR. According to these results, we deleted the predicted sequence which could not bind with StsR and listed them in Table 1.

**Table 1:** The identified putative StsR binding sequences in the promoters of selected transporter operons.

Function of operons	Genes	*p*-value	Matched sequence
PTS system mannitol-specific transporter	*smu.1182–smu.1185*	0.000835	TACAGTAGTTTTAGA
PTS system lactose-specific transporter	*smu.1488–smu.1498*	0.000254	AAAAATTATATTTTA,
Maltose ABC transporter	*smu.1566-smu.1571*	0.000182	TATTATTGTAACAAA
Multiple sugar-binding ABC transporter	*smu.876–smu.882*	0.000182	TAAAATAGTATTGTT
PTS system mannose-specific transporter	*smu.1956–smu.1961*	0.00105	AAAAAATGTAAAATA

### Characterization of the DNA Motif Recognized by StsR

**FIGURE 11:**
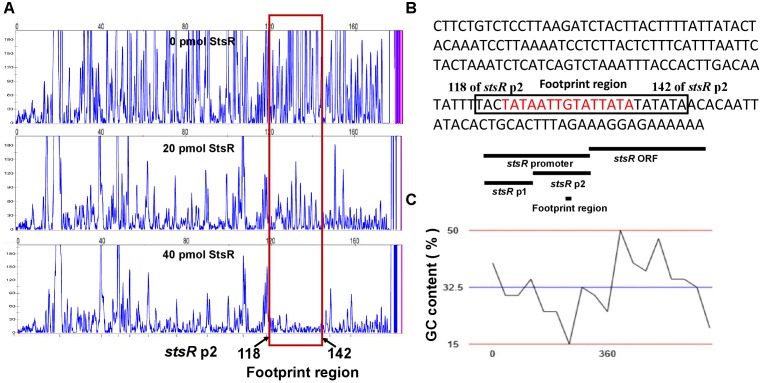
DNase I footprinting assays for the identification of DNA motif bound by StsR. **(A)** DNase I footprinting results. Electropherograms indicate the protected patterns of the *stsR* p2 promoter after digestion with DNase I following incubation with 0, 20, or 40 pmol StsR protein. **(B)** Nucleic acid sequence of *stsR* p2. The protected region is indicated with a black box, and the palindrome is marked with red. **(C)** The GC content of the DNA region from *stsR* promotor to *stsR* ORF is analyzed. The blue line showed the average GC content of this region is 32.5%.

**FIGURE 12:**
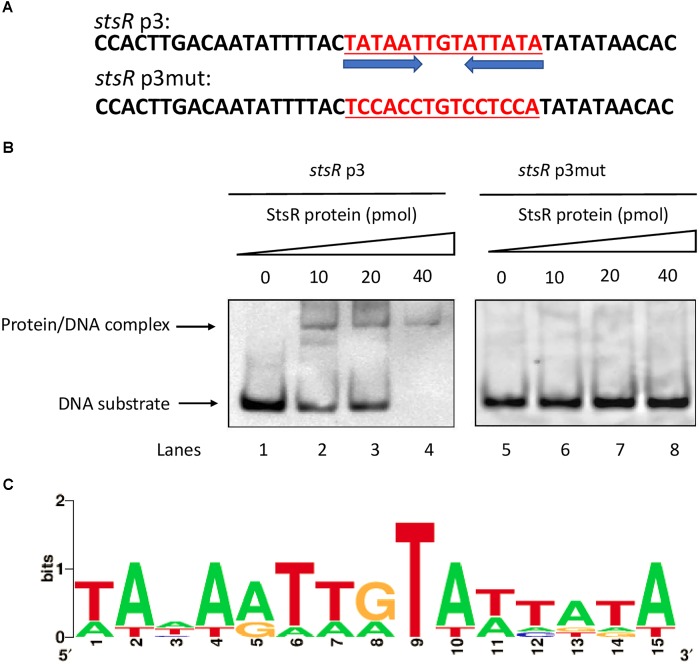
Identification of the conserved DNA motif bound by StsR. **(A)** Two DNA substrates containing the conserved palindrome sequences and mutant sequences were designed and synthesized. The conserved motif sequence is underlined. **(B)** EMSA assays for the DNA-binding activity of StsR on different substrates. Each of the two oligonucleotide substrates was mixed with 0, 10, 20, or 40 pmol protein. **(C)** Sequence logo for StsR binding motif was generated by WebLogo tool.

DNase I footprinting assays were conducted to determine the binding motif of StsR using the *stsR* p2 fragment labeled with FITC. As shown in Figure 11A, the region from 118 and 142 bp (relative to the *stsR* p2 fragment) was protected by StsR protein. This region covers a DNA motif 5′-TATAATTGTATTATA-3′, which is a short 15 bp palindrome (Figure 11B). Further GC content analysis of the StsR-DNA interaction region found in the DNase I footprinting assays was performed. We found that the region around binding motif 5′-TATAATTGTATTATA-3′ had at the lowest GC content of the entire region from *stsR* promotor to *stsR* ORF (Figure 11C). These results indicated that StsR bound to promoter regions with low GC content. We further confirmed that this DNA motif is essential for the recognition of StsR by designing a series of DNA substrates. As shown in Figure 12A, the *stsR* p3 fragment containing the 15 bp palindrome was labeled with FITC, and in the *stsR* p3mut fragment we changed several A/T bases to G/C to increase the GC content. We found that StsR was capable of binding with *stsR* p3, but was not incapable of binding to DNA substrates containing mutated motifs (*stsR* p3mut) (Figure 12B). These results clearly indicate that the DNA motif 5′-TATAATTGTATTATA-3′ is the binding site recognized by StsR.

To further investigate the conserved DNA binding motif for StsR, we used FIMO (Find Individual Motif Occurrences) to determine if the motif found by DNase I footprinting could also be detected in the promoter regions of StsR regulated genes. Based on the results of RNA-seq, we choose those operons that were predicted to encode sugar transport systems. The StsR binding motif 5′-TATAATTGTATTATA-3′ was matched with the predicted promoter sequences of sugar transport operons using FIMO (Grant et al., 2011). As shown in Table 1, the lower *p*-value means the better match between the DNA binding motif and the promoter regions. All the matched promoter regions identified had low GC content. These results were consistent with the DNase I footprinting results and the EMSA results, which indicated that StsR had a DNA binding preference for low GC regions. A further logo assay for the consensus sequence was conducted using the WebLogo tool^5^. A more general conserved motif for StsR recognition was mapped out (Figure 12C).

## Discussion

The biofilm formation and EPS production abilities of *S. mutans* are important for its virulence and the develop of dental caries. PTS systems, which are necessary for the transport and metabolism of sugar, have been shown to influence biofilm formation and bacterial virulence (Vadeboncoeur and Pelletier, 1997; Abranches et al., 2003, 2006; Loo et al., 2003). Previous studies have suggested a connection between sugar transport systems and the biofilm formation ability and EPS synthesis of *S. mutans* (Kawada-Matsuo et al., 2012; Moye et al., 2014a; Zeng et al., 2017). However, the regulatory mechanisms underlying this connection is still poorly understood. In the current study, we identified a GntR family transcription factor, named StsR. Knockout of *stsR* gene can significantly decrease the formation of biofilm and the production of EPS at early stage. Transcriptome analysis results suggest that the phenotypic changes caused by *stsR* deletion rely on its regulation of sugar transporter expression. Furthermore, we characterized the DNA binding motif of StsR with its own promoter using EMSA and DNase I footprinting assays and characterized the binding site of StsR with the promoter of the sugar transporter operons it regulates using bioinformatical method and verified with EMSA.

In *S. mutans*, the carbohydrate transporter systems contain at least five sugar ABC transport systems, 15 PTSs, and several sugar-specific multiprotein permeases known as enzyme II (Ajdic et al., 2002). The regulation of sugar metabolism in *S. mutans* primarily consists of the regulation of different transporter systems for sugar incorporation and the regulation of the synthesis of EPS. Based on our gene cluster analysis, inactivation of *stsR* resulted in the downregulation of a large number of genes and operons encoding mannitol-specific, lactose-specific, and mannose-specific PTS systems and genes involved with the multiple sugar-binding ABC transporter and maltose ABC transporter (Figure 9). Interestingly, the expression levels of *gtfs*, which encoded the main EPS synthesis enzymes in *S. mutans*, were not significantly changed in the *stsR* deletion strain (data not shown). These results suggest that the deletion of *stsR* could lead to a decrease in sugar uptake, which would further cause a decrease in the energy and substrate sources for *S. mutans* growth and sugar metabolism. These results could also explain the growth delay and the decrease in early biofilm formation and EPS synthesis caused by the deletion of *stsR* in *S. mutans.* However, this hypothesis needs further exploration.

Transcription factors play important roles in the regulation of sugar transport and metabolism in *S. mutans* virulence (Ajdic et al., 2002). Different regulation factors were involved in the complex regulation of sugar metabolism and response to different environmental stimuli. For instance, the regulation of carbohydrate catabolite repression (CCR) and expression of EII sugar transport permease genes from multiple PTS systems are regulated by transcription factor CcpA (Abranches et al., 2008). On the other hand, CCR is primarily governed by the PTS systems, which sense environmental carbohydrates and modulate the expression of genes involved in sugar catabolism. Moreover, seryl-phosphorylated HPr regulates CcpA-independent CCR in conjunction with PTS permeases in *S. mutans* to prioritize carbohydrate utilization by modulating sugar transport and the transcription of catabolic operons (Zeng and Burne, 2010; Moye et al., 2014b).

In addition to CcpA and HPr mentioned above, sugar transport systems are also co-regulated by multiple transcription factors in *S. mutans*. The sugar-binding ABC transporter genes and PTS system operons regulated by StsR have been reported to be regulated by other transcription factors and systems. For example, the MsmR protein has been reported as an MSM (multiple sugar metabolism) operon regulatory protein that regulates the expression of multiple sugar-binding ABC transporters (Russell et al., 1992; McLaughlin and Ferretti, 1996; Ajdic et al., 2002). The mannose PTS system is also regulated by ManL (Moye et al., 2014c), FruR, and EII^Man^ (Zeng et al., 2017). The mannitol-specific PTS system is also regulated by MtlR (Honeyman and Curtiss, 2000). The lactose-specific PTS system is also regulated by LacR (Rosey and Stewart, 1992). Since these PTS system genes are regulated by numerous regulatory systems in addition to StsR, the downregulation of these genes caused by StsR might be compensated by other regulation factors. These evidences could explain why the delays in growth rate, biofilm formation, and EPS synthesis caused by *stsR* deletion in *S. mutans* disappeared as time went on. Moreover, these could also explain why the growth curves were different when added different carbohydrates. Since the transporters of sucrose might be downregulated much more than the transporters of glucose and lactose or the downregulated of the transporters of glucose and lactose caused by StsR could be compensated by other regulation systems and factors. However, this speculation and the possible interactions and cross-talks among the different sugar metabolism regulatory systems require further study.

The transcription factor StsR identified in this study belongs to GntR family. In *S. mutans*, NagR, one of the seven GntR transcription factors reported, has been shown to differentially regulates the expression of the *glmS* and *nagAB* genes, which are required for amino sugar metabolism and the synthesis and catabolism of GlcN and GlcNAc (Zeng and Burne, 2015). In the present study, we found that StsR regulated the expression of various of PTS systems and further impacted the biofilm formation and EPS production. Our findings expanded the physiological functions and provided new information of GntR family transcription factors in this bacterium.

GntR family members are typically two domain proteins with a smaller N-terminus domain (NTD) with conserved architecture of winged-helix-turn-helix (wHTH) for DNA binding and a larger C-terminus domain (CTD) or the effector binding domain which is also involved in oligomerization (Finn et al., 2014; Jain, 2015). In *P. aeruginosa*, GntR has been reported to employ an effector-mediated de-repression mechanism, which means this regulator is promoter-bound in the absence of ligand. The binding of the metabolite regulated by this protein to the DNA/protein complex causes the release of GntR. This permits the RNA polymerase access to the promoter, allowing for transcription (Daddaoua et al., 2017). In *S. mutans*, the StsR protein identified in this study might also be regulated by its effector in response to specific environmental stimuli. Moreover, we found that the conserved DNA motif bound by StsR was a palindromic sequence with low GC content, which suggested that StsR preferred binding to DNA regions with low GC content and might function as dimer. These findings are consistent with the findings of previous studies that most GntR members are homodimers and recognize palindromic or pseudo-palindromic sequences of DNA that are A/T rich (Jain, 2015; Suvorova et al., 2015). Currently, studies are under way to identify the StsR ligand and to explore the detailed biochemical characteristics of StsR.

In summary, our findings suggest that *S. mutans* StsR is a novel GntR family transcription factor that regulates the expression of multiple sugar transport operons, and affects early biofilm formation and EPS synthesis, which are important virulence traits of *S. mutans* involved in the pathogenesis of dental caries. This report also provides important information for understanding the complex regulatory network of sugar metabolism and biofilm formation in *S. mutans*.

## Author Contributions

YL and JL conceived and supervised the studies and wrote the manuscript. ZL performed all experiments and wrote the manuscript. ZL, ZX, and JZ carried out bioinformatics analysis. YL, JL, ZL, ZX, and JZ analyzed the data. The manuscript had been reviewed by all authors before submission.

## Conflict of Interest Statement

The authors declare that the research was conducted in the absence of any commercial or financial relationships that could be construed as a potential conflict of interest.

## Footnotes

1 http://www.imageanalysis.dk

2 http://primer3.ut.ee/

3 http://david.abcc.ncifcrf.gov/

4 http://www.geneontology.org/

5 http://weblogo.berkeley.edu/logo.cgi

## Supplementary Material

FIGURE S1 Effects of GntR family genes deletion on biofilm formation and production of glucans in *S. mutans*. **(A)** *S. mutans* strains were cultured in BM supplemented with 1% sucrose. Then, the biofilm biomass was determined by CV staining. Data from three biological replicates were averaged, and the statistical significance between the *S. mutans* mutants and wild-type was determined by Student’s *t*-test. Error bars represent standard deviations based on results from at least three biological replicates. ^∗^Indicates a significance of *P* < 0.05. ^∗∗^Indicates a significance of *P* < 0.01. **(B)** The amount of water insoluble glucans in the biofilms of *S. mutans* UA159 and mutant strains were quantified using the phenol-sulfuric acid method and calculated according to the standard curve. Error bars represent standard deviations based on results from at least three biological replicates. ^∗^Indicates a significance of *P* < 0.05. ^∗∗^Indicates a significance of *P* < 0.01.

FIGURE S2 Determination of biofilm formation curves of *S. mutans* UA159, *S. mutans* Δ*stsR*, and Δ*stsR/pDL278-stsR*. *S. mutans* was cultured in BM supplemented with 1% sucrose for 2, 4, 8, 12, and 24 h. The biofilm biomass was determined by CV staining method. Data from three biological replicates were averaged, and the statistical significance between the *stsR* mutant, wild-type, and complement strain was determined by Student’s *t*-test. Error bars represent standard deviations based on results from at least three biological replicates. ^∗∗^Indicates a significance of *P* < 0.01.

FIGURE S3 Quantitative RT-PCR assays for the relative expression levels of respective genes in the down regulated sugar transporters in *S. mutans* UA159 and *S. mutans* Δ*stsR*. The experiments were carried out as described in Experimental procedures. All target genes were amplified using specific primers. Different gene expressions were normalized to the levels of 16S rRNA gene transcripts, and the folds of expression change were calculated.

FIGURE S4 Binding of StsR to the predicted promoter sequences of sugar transport operons determined by FIMO. A total of 20 pmol of DNA substrates were incubated with different amounts of StsR protein. EMSA results for StsR binding to the lactose-specific PTS system promoter gene (lanes 1–4), the mannose-specific PTS system promoter (lanes 4–8), the mannitol-specific PTS system promoter (lanes 9–12), the maltose ABC transporter promoter (lanes 13–16) and the multiple sugar-binding ABC transporter promoter (lanes 17–20).

TABLE S1 Bacterial strains, plasmids, and primers used in this study.

TABLE S2 List of genes upregulated greater than 2.0-fold.

TABLE S3 List of genes downregulated greater than 2.0-fold.

TABLE S4 Gene-annotation enrichment analysis of differentially regulated genes in KEGG pathway using DAVID tools (*P* < 0.05).

TABLE S5 Gene-annotation enrichment analysis of differentially regulated genes in GO terms using DAVID tools (*P* < 0.05).

TABLE S6 List of genes expression in the *stsR* mutant strain.
