# Supplementary figures and images for: A GntR Family Transcription Factor in Streptococcus mutans Regulates Biofilm Formation and Expression of Multiple Sugar Transporter Genes
Source: Front Microbiol. 2019 Jan 14;9:3224. doi: 10.3389/fmicb.2018.03224 (PMC6340165; doi:10.3389/fmicb.2018.03224)

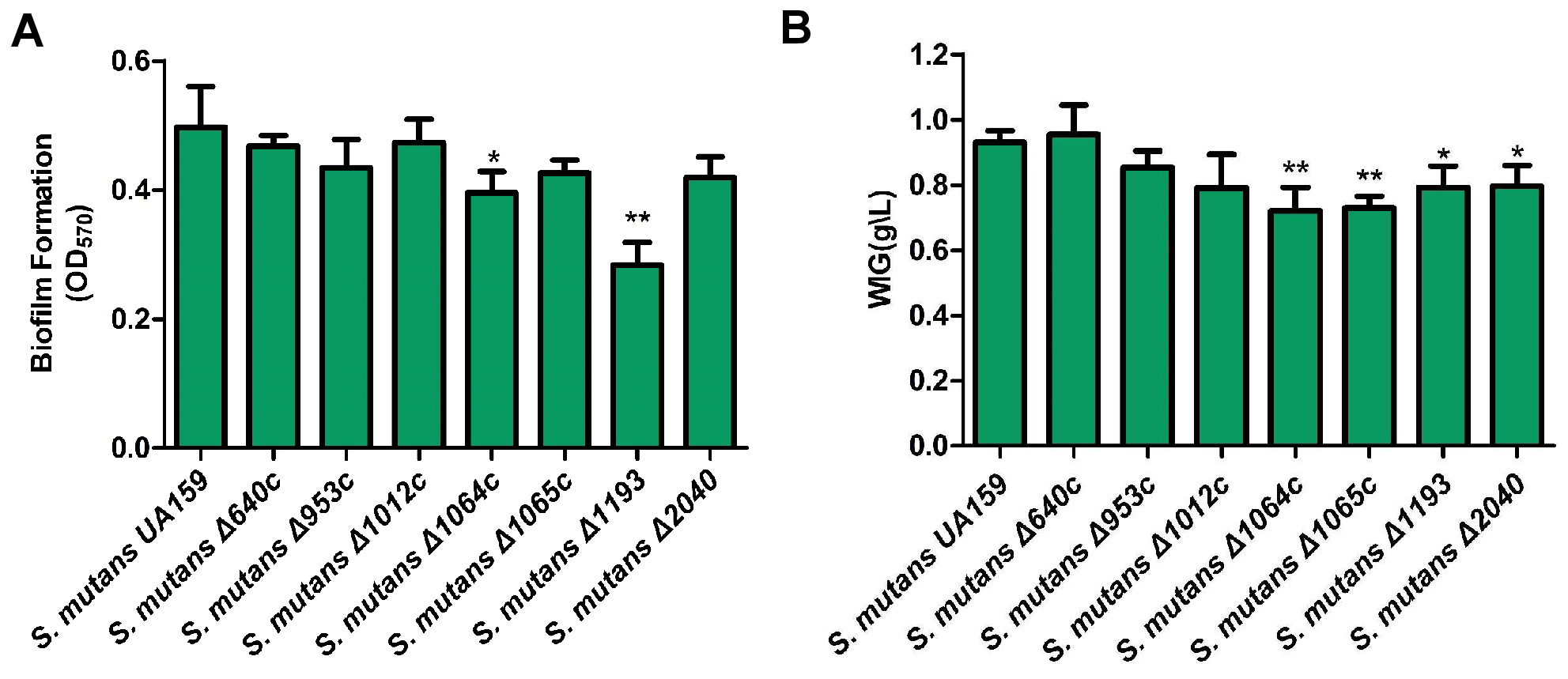

Supplement: FIGURE S1 — Effects of GntR family genes deletion on biofilm formation and production of glucans in S. mutans. (A) S. mutans strains were cultured in BM supplemented with 1% sucrose. Then, the biofilm biomass was determined by CV staining. Data from three biological replicates were averaged, and the statistical significance between the S. mutans mutants and wild-type was determined by Student’s t-test. Error bars represent standard deviations based on results from at least three biological replicates. ∗Indicates a significance of P < 0.05. ∗∗Indicates a significance of P < 0.01. (B) The amount of water insoluble glucans in the biofilms of S. mutans UA159 and mutant strains were quantified using the phenol-sulfuric acid method and calculated according to the standard curve. Error bars represent standard deviations based on results from at least three biological replicates. ∗Indicates a significance of P < 0.05. ∗∗Indicates a significance of P < 0.01. [file Image_1.TIF]

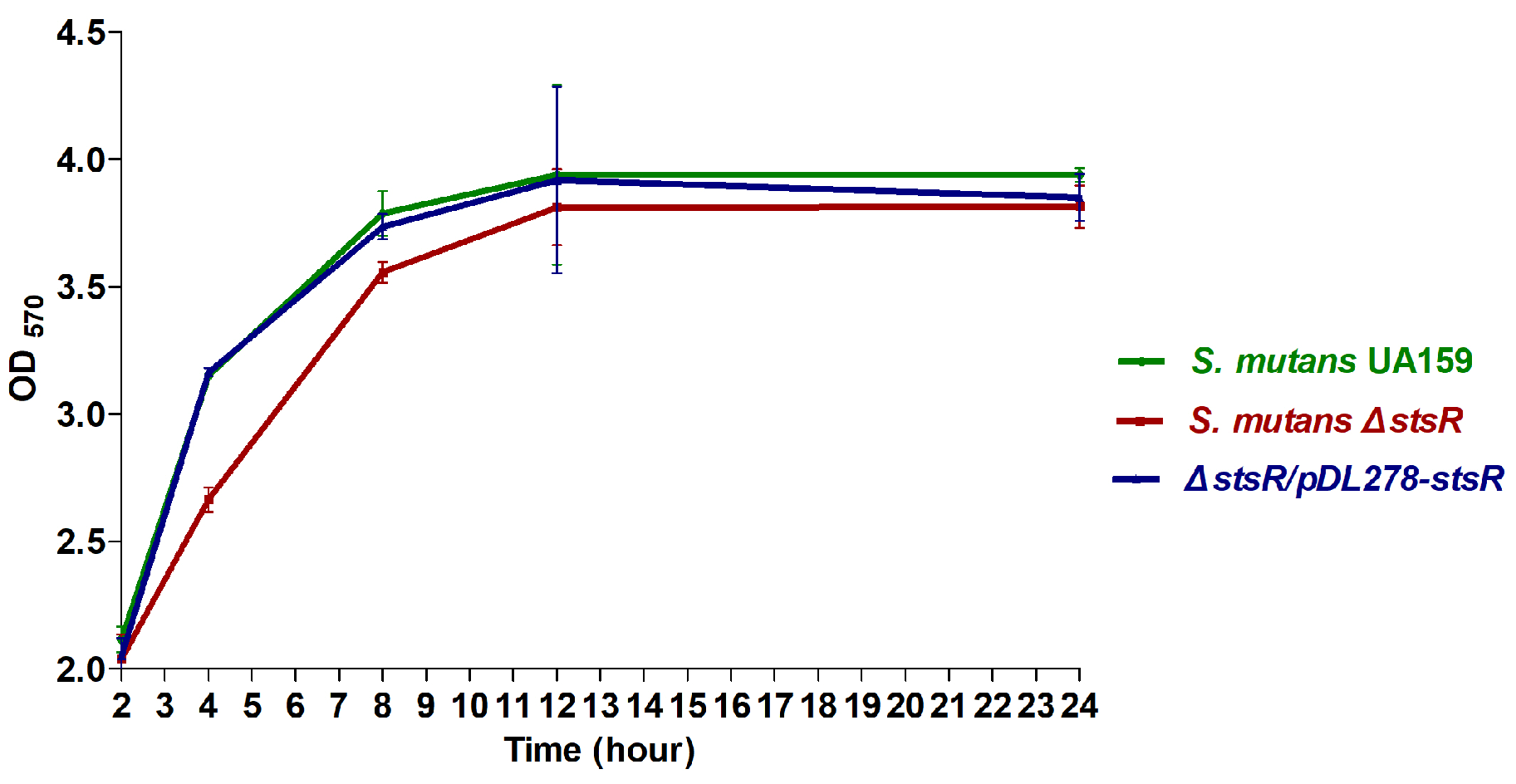

Supplement: FIGURE S2 — Determination of biofilm formation curves of S. mutans UA159, S. mutans ΔstsR, and ΔstsR/pDL278-stsR. S. mutans was cultured in BM supplemented with 1% sucrose for 2, 4, 8, 12, and 24 h. The biofilm biomass was determined by CV staining method. Data from three biological replicates were averaged, and the statistical significance between the stsR mutant, wild-type, and complement strain was determined by Student’s t-test. Error bars represent standard deviations based on results from at least three biological replicates. ∗∗Indicates a significance of P < 0.01. [file Image_2.TIF]

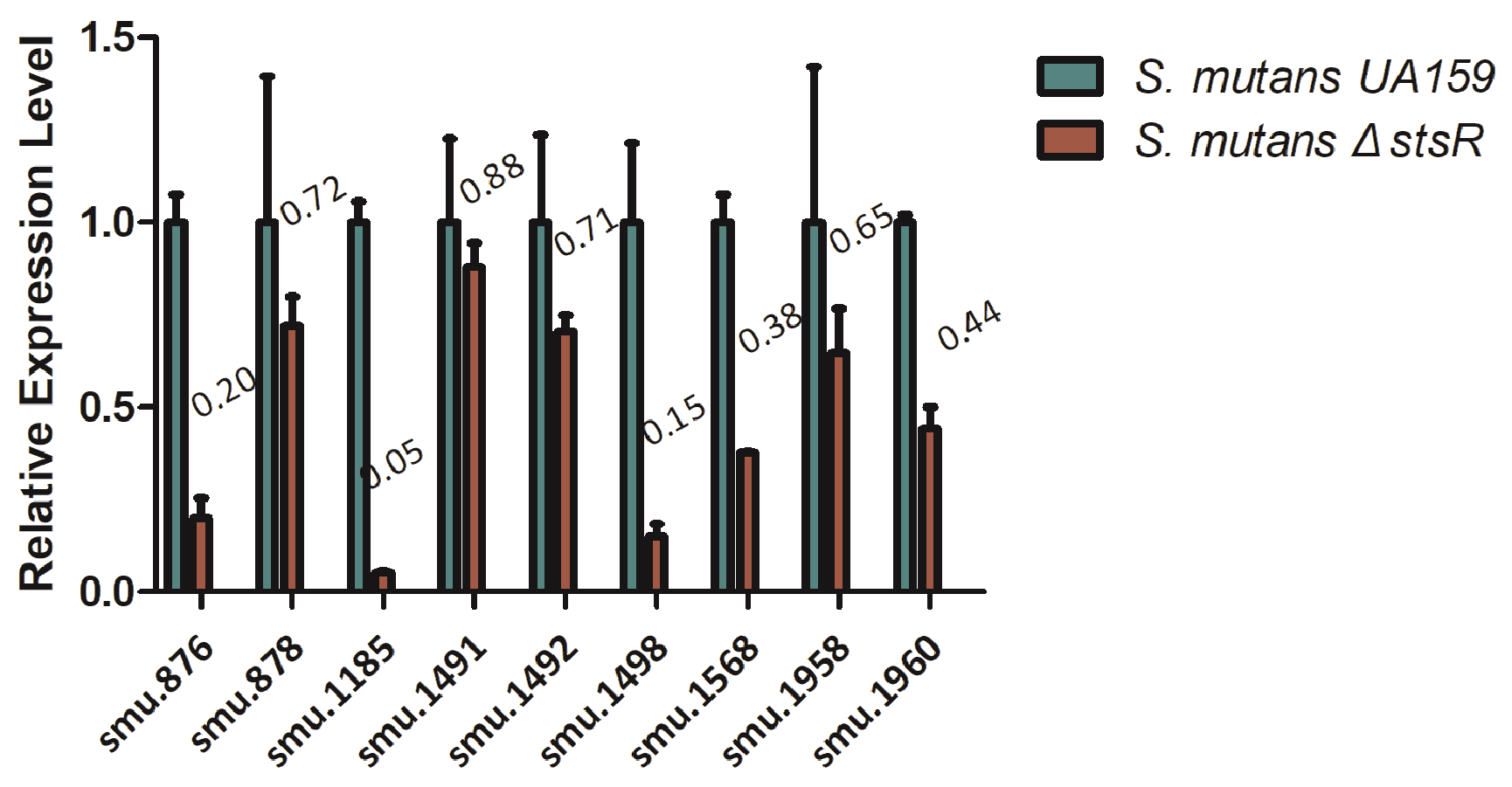

Supplement: FIGURE S3 — Quantitative RT-PCR assays for the relative expression levels of respective genes in the down regulated sugar transporters in S. mutans UA159 and S. mutans ΔstsR. The experiments were carried out as described in Experimental procedures. All target genes were amplified using specific primers. Different gene expressions were normalized to the levels of 16S rRNA gene transcripts, and the folds of expression change were calculated. [file Image_3.TIF]

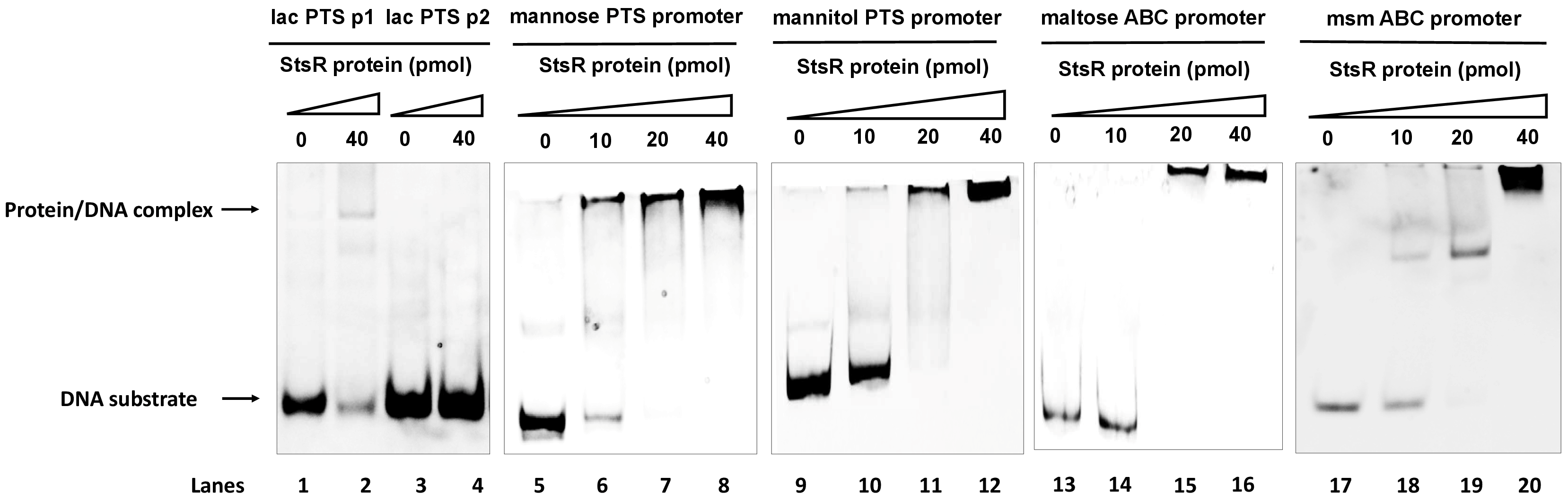

Supplement: FIGURE S4 — Binding of StsR to the predicted promoter sequences of sugar transport operons determined by FIMO. A total of 20 pmol of DNA substrates were incubated with different amounts of StsR protein. EMSA results for StsR binding to the lactose-specific PTS system promoter gene (lanes 1–4), the mannose-specific PTS system promoter (lanes 4–8), the mannitol-specific PTS system promoter (lanes 9–12), the maltose ABC transporter promoter (lanes 13–16) and the multiple sugar-binding ABC transporter promoter (lanes 17–20). [file Image_4.TIF]
